# Traditional Sporting Games as Emotional Communities: The Case of Alcover and Moll’s Catalan–Valencian–Balearic Dictionary

**DOI:** 10.3389/fpsyg.2020.582783

**Published:** 2021-01-18

**Authors:** Antoni Costes, Jaume March-Llanes, Verónica Muñoz-Arroyave, Sabrine Damian-Silva, Rafael Luchoro-Parrilla, Cristòfol Salas-Santandreu, Miguel Pic, Pere Lavega-Burgués

**Affiliations:** ^1^Motor Action Research Group (GIAM), National Institute of Physical Education of Catalonia (INEFC), INDEST, University of Lleida, Lleida, Spain; ^2^NeuroPGA Research Group, Department of Psychology, University of Lleida, Lleida, Spain; ^3^Motor Action Research Group (GIAM), Institute of Sport, Tourism, and Service, South Ural State University, Chelyabinsk, Russia

**Keywords:** intangible cultural heritage, motor praxeology, ethnomotricity, sustainability, relational wellbeing

## Abstract

Learning to live together is the central concern of education everywhere in the world (UNESCO). Traditional sporting games (TSGs) provide interpersonal experiences that shape miniature communities charged with emotional meanings. The objective of this study was to analyze the ethnomotor features of TSG (relationship between the internal characteristics of the TSG and sociocultural variables) in three Catalan-speaking Autonomous Communities and to interpret them for constructing emotional communities. The study followed a phenomenological-interpretative paradigm. The identification of TSG was done by a hermeneutic methodological approach by using an exhaustive exploratory documentary research. We studied 503 games collected in the Dictionary Català-Valencià-Balear de Alcover and Moll (1926–1963). Instruments and procedure: A database was built up with information about the internal and external logic of the games. The validity of the information was confirmed by means of a concordance test between the researchers. Data processing was carried out by means of classification trees (inferential level), identifying the predictive variables of the types of TSG. Most of the TSGs were sociomotor games (*n* = 405/503; 80.5%). The classification tree identified four explanatory variables. Three variables were internal traits (body contact, material, and score), and one variable corresponded to external logic (age). Features of the TSG of the Catalan-speaking Autonomous Communities build original emotional communities. The ethnomotor regularities triggered emotional experiences associated with pleasure for (a) living together (predominance of sociomotor games); (b) domesticating of aggressiveness over opponents (different motor licit aggressiveness); (c) developing sustainability (presence and absence of objects from the surrounding environment); (d) educating the competition (games with and without final score); and (e) interpersonal well-being based on the community (transmission of ludic culture from children to young TSG).

## Introduction

Distinguished international organizations such as UNESCO have repeatedly stated that the main concern of education anywhere in the world is learning to live together (Yan Fang et al., 2014). Before reaching adulthood, people share a multitude of social experiences in formal (learning recognized by each country’s education system that leads to certificates and qualifications), non-formal (additional learning, optional to formal), and informal (learning in daily life, in the family, in communities, without planning) educational scenarios (UNESCO, 2012).

In the context of informal learning, the traditional sporting game (TSG) is one of the main languages of socialization. In contrast to sport, the TSGs are confrontational situations whose rules are established by the participants themselves, according to local tradition (Parlebas, 2016). On the other hand, a sport is codified by a central institution (federation) with the intention of extending it to the whole world.

In informal or non-formal educational environments, societies have originated a remarkable diversity of playful experiences, unique methods of entering into relationships with others, and ways of following social rules. After school, during weekends and holidays, children and young people are used to playing with other people, weaving very special bonds of friendship and interpersonal relationships. In these scenarios, the TSG is a miniature society, where the protagonists learn to live together in the community (Parlebas, 2001). The TSGs anchor aspects of culture and weave bodily techniques or habitus of attitudes, gestures, and social preferences (Mauss, 1936; Geertz, 1983).

The relationships that each game activates help to build small communities, integrated by people who share emotions, beliefs, and responsibilities (McMillan and Chavis, 1986; Treitler et al., 2018). Thus, TSGs create what Rosenwein (2006) calls emotional communities, since their protagonists share emotional states that arise from the democratic dialogue of the rules of the game.

Playing a game means being involved in a social exchange of emotions with the other players (Lavega et al., 2014). Many TSGs establish rituals to distribute the roles of the pursuer (it) or captain, as well as to decide the order of intervention. In this first phase, people begin to feel that they share an emotional intrigue about how to start the game. Previous studies reveal that playing TSG elicits intense emotions, for example when players agree on a team strategy; when they share ludic actions as chasing, capturing, or running away from an opponent; or when they share the victory or complain about the defeat (Etxebeste et al., 2014; Duran and Costes, 2018). Traditionally, children and young people spend a large part of their leisure playing TSGs (Etxebeste, 2012; Etxebeste et al., 2015; Lavega-Burgués and Navarro-Adelantado, 2015; Parlebas, 2016).

Such persistent playful involvement helped each group of players to have their own particular values, modes of feeling, and ways to express playful feelings (Rosenwein, 2016). Emotional communities are therefore the process of viewing a social group by the way it assesses emotions and according to the norms it follows for how emotions should be expressed. In other words, any group of people with common interests and goals can be called an emotional community. As Rosenwein (2006) highlights, each person may belong to several emotional communities, simultaneously or successively. In the course of one’s life, we can move from one emotional community to another and be in several communities at the same time. This means that during childhood and youth, people participate in the construction of a ludic emotional community (Rosenwein, 2006).

Social in nature, TSGs become the mirror of the predominant characteristics of the communities, reflecting through their own rules the expression of social rules. Thus, it is necessary to reveal the unique nature of the rules of the game in order to identify the specificity of the ways of interaction.

The science of motor action offers scientific fundamentals to study the internal logic of the TSG, that is, the properties contained in the rules, independently of the characteristics of their protagonists (Parlebas, 2001). The internal logic is defined by “the system of relevant traits of a motor situation and the subsequent consequences in the completion of the corresponding motor action” (Parlebas, 2001, p. 216).

When playing a game, players respond to relationships established by their internal logic, that is, (a) relationship with space: participation in a stable surface (without uncertainty) or unstable surface (with uncertainties); (b) relationship with material: presence or absence of objects; (c) relationship with time: way of ending (presence or absence of a final score that establishes winners and losers); and (d) relationship with others (type of motor interaction with the other participants, or for instance, the allowed degree of intensity in/with body contact).

Regarding relationship to others, two categories of TSG are identified: (a) psychomotor TSG, where the person plays without partners or opponents as in the game of *Quernet* (knock down an almond pillar by throwing a much bigger almond), and (b) sociomotor TSG (two or more people cooperate to reach a common goal); e.g., in the game of *Molí* (two children join hands and turn around without moving their feet from the ground and increasing the speed of the turn); of opposition (the player opposes one or more opponents; e.g., in the game of *Estiracabells*, the children dispute a ball to take it to a designated place); or of cooperation–opposition (with motor interactions with partners and opponents; e.g., in the game of *Romaní-romanà*, one team chases another to make them prisoners).

The internal logic of the game also determines the level of motor interactions allowed by the rules. Thus, depending on the distance of confrontation between the opponents and the authorized body contact (Parlebas, 2017), different levels of motor aggression may be triggered (Collard, 2004). Aggression may be legal, permitted by the rules of the game (e.g., hitting an opponent in a fighting game), and should not be confused with aggression or physical violence involving the use of force (Elias and Dunning, 1986), which is illegal and sanctioned. Physical contact can also take place in cooperative games; however, it will not be logically associated with motor aggressiveness.

In addition to having a system of rules, TSGs have a direct relationship with the local culture to be included in any sociocultural interpretation. Motor praxeology offers the concept of ethnomotricity to connect the internal traits of the games (internal logic) with variables external to the rules (external logic) or social and cultural conditions: characteristics of the players (sex; age); playing areas (location and conditioning); times of practice (with or without a calendar); and provenance of the objects (from the domestic, natural, or purchased environments) (Etxebeste et al., 2015).

Other ethnomotor studies in different countries show the mirror function of local culture [e.g., Etxebeste (2012) illustrates the connection of the TSGs with the traditional culture in the Basque Country; Lavega-Burgués and Navarro-Adelantado (2015) reveal the ethnomotor features of the TSG in Spain described by Rodrigo Caro; Parlebas (2016) points out the connection of the TSG with the cultural features of societies]. The effects of the TSG on the emotional states of members of the ludic community have also been noted (Lavega et al., 2014).

From this perspective, the aim of this research was to reveal the ethnomotor traits of the TSG of three autonomous communities (regions) of Spain and their possible link with the production of singular emotional communities.

## Methods

The design of the research was based on the phenomenological-interpretative paradigm (González-Monteagudo, 2001). In the identification phase of the TSG, the study followed a hermeneutic methodological approach (Pérez, 2011) by using an exhaustive exploratory documentary research.

In the process of classifying the TSG, we carried out a content analysis, which is typical of qualitative methodology, using data and research triangulation.

From a methodological point of view, the ethnomotor research of TSG performed in other historical periods has to face the limitations offered by written sources. Often, there is a problem with the quality of information described about the rules, habits, and customs associated with TSG. This limitation is even more severe when the work is written by a single author. To answer these problems, this study based on the “Diccionari Català-Valencià-Balear” (DCVB) has taken into account the following methodological considerations: (a) The same methodological procedure has been followed as in other previous researches, for instance, Parlebas, 2003; Etxebeste, 2012; Lavega-Burgués and Navarro-Adelantado, 2015). (b) The DCVB dictionary is considered as one of the first etymological dictionaries of the Romance languages elaborated with a contrasted scientific procedure (Domènech and López, 1999; Perea, 2012) involving a team of eminent philologists and more than 5,000 informers; therefore, the scientific lexicographic community grants objectivity and reliability to the contents of the work. (c) Many games have been identified in several localities, which confirms their presence and also complements the description of the rules and their sociocultural aspects. (d) When there were doubts in any game to interpret its rules, we consulted the books of the philologist, lexicographer, and etymologist Corominas (1980–1981) and of the ethnologist and folklorist Amades (1982–1983) and the specific books of games in Maspons (1928) and Vidal (1893).

### Sample

We studied 503 TSGs performed by different ages, mostly by children and young people and by both genders. The TSGs were located in Catalonia, Valencia, the Balearic Islands, and the South of France (formerly known as Northern Catalonia), described in the Dictionary DCV written by the authors Alcover and Moll (1968, 2018). This work, composed by 10 volumes, has 9,850 pages that make up 160,000 articles or entries describing the meaning of all the words, among them the games played until the end of the XIX century and the beginning of the XX century, which were used in the diverse dialectal modalities of the Catalan language.

### Instruments and Procedure

A database was elaborated, and the variables referred to the internal logic were identified for each TSG: (a) authorized body contact against the opponent, (b) material, and (c) result (outcome), and to the external logic: (a) region; (b) origin of the material, (c) gender, (d) age, (e) location zones, (f) conditioning zones, and (g) calendar. To ensure the quality of the registers, four expert compilers were recruited (with more than 10 years of training in motor praxiology). A three-phase procedure was followed (Arana et al., 2016): (i) theoretical training and construction by consensus of an *ad hoc* tool, based on internal and external logic; (ii) theoretical and practical training of observers with examples and counter-examples for classification; and (iii) classification of all the TSGs independently and without interference among the observers, when the tool was prepared. This procedure (Anguera, 2003) was carried out twice (intra-observer) with a distance of 1 month, by the four observers (interobserver). The interobserver and intra-observer Spearman’s (=1.00), Pearson (=1.00), and Cohen’s kappa (=1.00) (Cohen, 1960; Lapresa et al., 2020) were used to ensure the data quality for analysis.

### Analysis of the Data

Statistical analysis was performed by means of classification trees (Lavega et al., 2014; Lavega-Burgués et al., 2020b) at the inferential level (Pearson’s chi-square) identifying the predictive variables (related to internal logic and to external logic) of the types of games according to the motor interaction they generate. We used the tree growth method known as CHAID (Chi-squared Automatic Interaction Detector, implemented in SPSS^TM^ 26, Answer Tree©). We followed a cross-validation system with a stopping rule of three maximum levels, with 50 being the minimum number of elements at the terminal nodes.

## Results

Regarding the total number of TSGs identified in the dictionary (*n* = 503), the majority corresponded to socio-motor situations (opposition games, cooperation, and cooperation–opposition games; *n* = 405; 80.5%), with a predominance of opposition games (*n* = 234; 46.5%).

**FIGURE 1:**
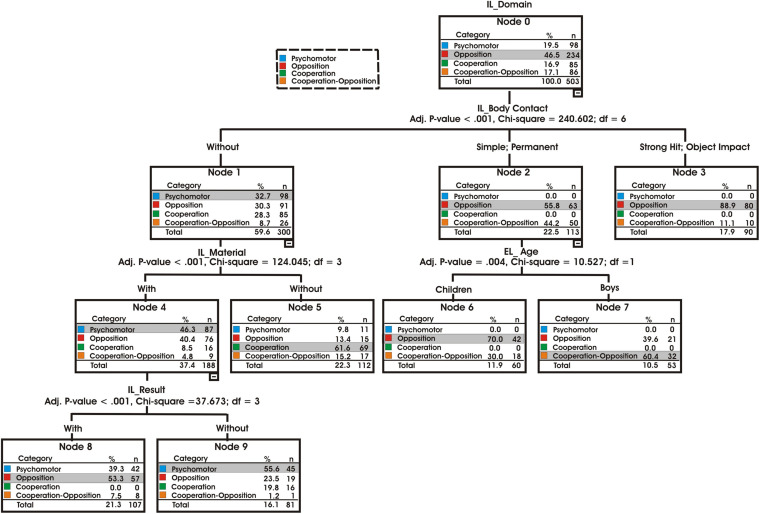
Classification tree: predictive ethnomotor variables of Catalan-speaking TSG.

The classification tree identified four explanatory variables of the games: Three variables corresponding to internal logic (body contact, material, and result) and one variable related to external logic (age) (see Figure 1). The overall classification accuracy of the model (CHAID algorithm) was 64.6%.

Among the characteristics of the internal logic, body contact with the opponents (relationship with the others) turned out to be the most representative. There were significant differences (*p* < 0.001; χ^2^ = 240.602; *df* = 6) between the three types of body contact. Most of the games were played without contact with the opponents (node 1) (*n* = 300; 59.6%).

Traditional sporting games with body contact (*n* = 113; 22.5%) were played with simple and permanent contact (node 2), represented exclusively by games with opposition (*n* = 63; 55.8%), TSGs with opposition and cooperation (*n* = 50; 44.2%), and 17.9% (*n* = 90) with strong impact or object contact against the opponents (node 3) were represented mainly by opposition games (*n* = 80; 88.9%).

In most cases, when games are played without contact of the opponents’ bodies (node 5), two predictive variables of internal logic were identified: the relationship with the material and with time (result) (*p* < 0.001; χ^2^ = 124.046; *df* = 3). Analysis of the “material” variable showed that, among these games without body contact, object games were the most prevalent (*n* = 188; 37.4%) mainly in psychomotor games (*n* = 87; 46.3%) and opposition games (*n* = 76; 40.4%).

Games without objects were less present (*n* = 112; 22.3%) than games with objects (*p* < 0.001; χ^2^ = 124.045; *df* = 3), represented mainly by cooperative games (*n* = 69; 61.6%).

Finally, in the TSG with objects the predictive variable “result” (outcome) showed that the TSGs with result or outcome were significantly predominant (*p* < 0.001; χ^2^ = 37.673; *df* = 3) and above all present in opposition games (*n* = 57; 53.3%). The TSGs without outcome corresponded mainly to psychomotor activities (*n* = 45; 55.6%).

In TSGs with simple or permanent contact, age was the external predictor variable. Significant differences were found (*p* < 0.004; χ^2^ = 10.527; *df* = 1). Children (*n* = 60; 11.9%) preferred opposition games (*n* = 42; 70%), and young people (*n* = 53; 10.5%) preferred cooperation-opposition games (*n* = 32; 60.4%).

## Discussion

The aim of this research was to reveal the ethnomotor traits of the TSGs of three Catalan-speaking autonomous communities and their possible connection with the construction of singular emotional communities.

The ethnomotor study, through the intelligibility of the internal and external characteristics of the Catalan-speaking TSG, revealed the production of possible emotional experiences.

### Games as a System of Social Interaction: A Socio-Emotional Well-Being

#### The Pleasure of Learning to Live Together

The different ways of motor relationship contained in the rules of the TSG show the type of social relationship they promote. The Catalan games are mostly sociomotor (*n* = 405/503; 80.5%).

Played with other players, TSGs are expressed in terms of motor interactions between participants, in particular through antagonistic socio-motor dynamics (46.5% are opposition games).

These characteristics allow a better understanding of the pleasant culture of playing together, of living together (see Table 1). Moreover, the triggering of decoding body signs, decision making, and motor strategy and the intense socio-affective dynamics caused by these oppositional situations create, at the same time, consequent emotional communities. This, in a possible intervention in formal education, presents a greater intelligibility of the educational action.

**TABLE 1:** The social construction of emotional communities through Catalan-speaking traditional sporting games.

**Key features of Catalan-speaking traditional sporting games**		
**Ethnomotricity**	**Educational and cultural values**	**Emotional values internal logic**
Internal logic: Relationship to others, material, time	Culture of social and environmental respect	Socio-emotional well-being
External logic: Age: young population	Culture of intergenerational transmission of heritage	Community interpersonal well-being

#### Learning to Domesticate Aggressiveness

Among the ten internal and external factors studied, bodily contact with opponents is the predominant trait of the internal logic of the inventoried games. Catalan games constitute a culture without adverse body contact (59.6%), that is, without illicit motor aggressiveness (Collard, 2004; Dugas et al., 2016). This trait is clearly present in opposition games (30.3%) as in *Catarroja* (the players hide and another person must guess where they are hiding, and he/she discovers them by saying their name).

When the games allow body contact, simple or permanent contact is preferred (22.55%) (moderate motor aggressiveness (Collard, 2004), mainly within opposition games (55.8%) As in *cat and mouse* (the cat followed the mouse to capture it with a simple touch).

High-impact or strong-impact games with objects (intense motor aggressiveness, Collard, 2004) represent a minority (17.9%) and are mostly composed of opposition games (88.9%) such as *Cordeta to amagar* (the player who finds a hidden rope chases the others and hits them with it).

The varied internal logic in this group of games teaches the protagonists to share legal motor aggressive actions of different intensity (Collard, 2004) and to regulate negative emotions in the face of reactions to frustration (Dollard et al., 1939; Berkowitz, 1989) or aggressive responses in a conflict (Dugas et al., 2016) or even to motor violence during the game (Elias and Dunning, 1986).

#### Developing Environmental Sustainability

The classification tree shows that games without body contact are played mainly with objects (37.4%), particularly in opposition games (40.4%) and psychomotor games (46.3%). Conversely, games performed without objects (22.3%) were mainly cooperative (61.6%).

Motor interactions in opposition games with material were carried out through contact between handcrafted objects (e.g., throwing a wooden marble on the opponents’ marble or balls, handkerchiefs, ropes, caps, or spinning tops).

In psychomotor games, there is no body contact; players take part side by side or in turns (e.g., in *Cinquetes*, different rhythmic actions are performed when throwing and collecting small stones).

All these games favor the development of environmental sustainability (respect for the natural environment) and social sustainability: participation (all the members are allowed to play), equality (the rules are the same for all the players), social cohesion (the community improves her internal relationships), and awareness of sustainability (the relationships have a long-term duration) (see Murphy, 2012). These dimensions are included in the sustainable development objectives in the Agenda 2030 adopted by the member states of the United Nations (2015).

The games with objects are authentic showcases of sustainable learning with a clear orientation toward environmental sustainability (Lavega-Burgués and Navarro-Adelantado, 2015). Unlike regulated sports, whose objects are bought and equal all over the world, playing with objects comes from a nearby environment (natural or domestic) (Parlebas, 2003). Before playing with these objects, it was necessary to perform two ecological actions: (a) searching for objects in the natural environment (e.g., stones, bones, branches, herbs, fruits, or reeds) or domestic environment (e.g., ropes, wheels, sacks, brooms, needles, shoes, and handkerchiefs) and (b) crafting this material (e.g., making stilts out of rope and paint cans). Recovering, recycling, and reusing were three common sustainable learning actions (see Table 1).

Other studies show that self-construction of playful objects arouses high levels of interest, enjoyment, and motivation (e.g., Méndez-Giménez et al., 2012) as would be the case with many traditional games.

#### Developing Social Sustainability

The second group of games without body contact over the opponents and without objects (*n* = 112) is mostly made up of cooperation games (*n* = 69; 69%). Examples are dance games, circle games, or choreographies, or Sant Joan de les *Cadenelles*: players holding hands move and sing. Other studies show that these TSG trigger intense interpersonal relationships which in turn activate very high states of relational and emotional well-being (e.g., Lavega et al., 2014).

According to the theory of contact (Allport, 1954), these cooperative games favor attitudes of positive social sustainability. By cooperating, the participants (a) act with equality of status within the group; (b) participate in the collective construction of the pact of rules and allow the playful community to support or sanction undesired conducts; (c) seek to achieve common objectives that unite them; and (d) intervene in genuine, deep, and intimate associations where energy, decision, emotion, and relationship are different dimensions of the same phenomenon of learning on social sustainability.

Once again, the presence of an exuberant playful diversity (Parlebas, 2001, 2003) is observed, which consolidates the learning oriented to sustainable well-being (see Table 1).

#### Learning to Interact With and Without Competition

Finally, for games without body contact and with material the following predictive variable is the result reached in the game. Two groups are identified: TSG with competition and without final score.

#### Learning to Compete

The games with result reached in the game (37.4%) are mostly competitive (*n* = 57; 53.3%). The internal logic of these games guides players to compare and classify their answers to proclaim winners and losers (Etxebeste et al., 2014; Parlebas, 2017). The struggle for victory emphasizes the tension in the motor actions of the rivals, although the use of objects allows the projection of all the playful energy on the other rival objects instead of over on their bodies (e.g., hitting the rivals’ spinning tops hard, even breaking them). These playful resources are resources/means of informal education aimed at learning to control motor aggressiveness.

In these games, the competition begins to be constructed by negotiating the group agreement of the rules. The players agree on the conditions in which they will face each other, which brings an interest in the activity from the very beginning of the game (Cumming et al., 2007; Etxebeste et al., 2014). The high emotional intensity of the opponent’s presence (e.g., Lavega et al., 2014; Duran and Costes, 2018) involves learning to establish a balance between enjoyment through interest in the process of the actions of one’s own game (task climate) or through motivation to want to dominate and exercise power over others (ego climate) (Lavega et al., 2014; Lavega-Burgués et al., 2020a).

#### Learning to Enjoy Without Comparison

When games are played without a final score, psychomotor games predominate (55.6%) over sociomotor games. Here, emotional excitement is directed toward the enjoyment of the process associated with the chaining of motor actions, often in a cyclical manner (Parlebas, 2001).

These games trigger emotional well-being when next conditions are present: (a) The possibility of reaching the objective on several occasions (e.g., throwing a spinning top so that it stays dancing for a while); (b) the intervention associated with having to make an effort (trying out different ways of throwing the top over and over again); and (c) being able to perform motor actions effectively (achieving the proposed objective) without having to compare oneself with others (e.g., Serna et al., 2017; Lavega-Burgués et al., 2020a).

### Play as a System of Social Integration: A Community-Based Interpersonal Well-Being

#### Constructing Emotional Communities in Formal and Informal Education

Among all the variables, the classification tree has identified only one external variable: the age associated with TSG with the presence of moderate or permanent body contact.

Children and young people play a similar percentage of games (children: *n* = 60; 11.9%; young people: *n* = 53; 10.5%). However, in children there is a superiority of opposition games (70%) over cooperation–opposition games (30%), while among young people this regularity is reversed (opposition games = 39.6%; cooperation–opposition games = 60.4%).

This finding is directly related to a fundamental learning process with the passage from child to young person, i.e., the adoption of the democratic pact with others and the acceptance of the community rule. Parlebas (1986) shows, through empirical studies, different stages of evolution in children’s attitudes to the rule. Up to the age of 11, children go through the stage of rejection of rules (2–6 years) and later (6–11 years) through the stage marked by egocentrism in which they gradually lean toward the adoption of a shared rule. In these stages, it is consistent that the games that predominate are those of opposition, in which each player has an individual objective, and he/she is the center of attention when facing the others. From the age of 11, young people accept the common agreement of the rules of the game and enjoy the pleasure of sharing common rules.

Traditional games play a key role in informal education, in playful experiences where the presence of an educator or an adult to lead the game was not necessary (UNESCO, 2012). In these informal contexts, playing teaches people to live together, to live in the community and to enjoy the pleasure of meeting others (Parlebas, 2003; Yan Fang et al., 2014), and to live together through processes of community normalization (Allport, 1954).

However, the TSG in the Catalan-speaking communities have particular rules, with different ethnomotor features from other communities. In these conditions, Catalan children and young people create body techniques in accordance with the values of the society to which they belong (Mauss, 1936; Parlebas, 2001).

Every playful action, every match in a traditional game is a masterful lesson in constructing emotional communities (McMillan and Chavis, 1986; Rosenwein, 2006). The raw material is served, so that, by playing, Catalan children and young people learn to establish norms of social conduct; they learn to construct models of interpersonal relationships in which everyone is equal, in contexts in which the rights and prohibitions established by the rules are the same for any participant (Etxebeste, 2012). This common norm is associated with a process of reciprocal concessions in which a corporal habitus of organic, cognitive, social, and eminently emotional affective nature is configured (Parlebas, 2001; Warnier, 2001). TSGs are real laboratories of interpersonal relationships that teach the actors to live in the community (Yan Fang et al., 2014).

**FIGURE 2:**
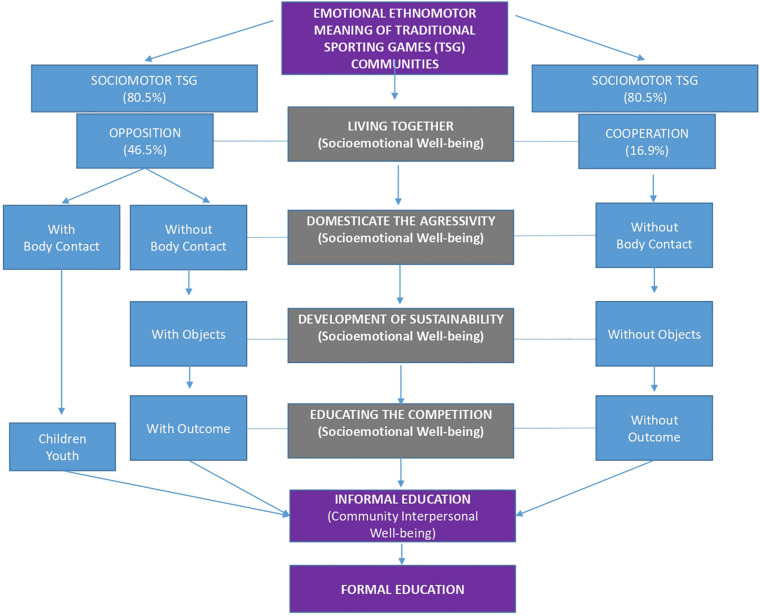
Ethnomotor meaning triggered by TSG in the process of constructing emotional communities.

The main habitat of the TSG is the informal education, in which the members of the community learn to agree on their rules and to choose which games to play at each moment; the way to start and end the game; and the challenges to be accomplished and what will be at risk. These are informal contexts with a high educational load that integrates the values of their community. For this reason, formal education should take advantage of this fundamental cultural knowledge to integrate it, mainly through quality physical education (Figure 2). In this way, it would be possible to respond to some of the major challenges agreed upon by ministers of the different countries that make up the United Nations, in the proposals of the agenda for sustainable development in 2030 (United Nations, 2015).

## Conclusion

This article presents an original way of analyzing the distinctive traits of more than 500 traditional games included in the Alcover and Moll (1968, 2018) dictionary. Each game is a piece of a puzzle, a miniature society (Parlebas, 2001) that, as if it were a mirror, reflects a clear connection of these TSG with the local culture of the Catalan-speaking communities.

As Warnier (2001) states, through motor practices such as TSG, there is the universal fact of becoming a human being. Being a citizen of the world means having access to the moral law that implies the relationship of oneself with others, in accordance with the social constrictions that each community establishes. The games, as Foucault (1982) would say, are procedures that serve to fix the individual and collective identity. Games are techniques of subjectivization and individual identity (Bizumic et al., 2009), in which playing means relating to other members of the community.

The ethnomotor traits of Catalan games of the period studied are above all sociomotor games, of opposition, without body contact, with objects, and with a final result. When the games are cooperative, they are also without body contact directed at the opponents, without an object and without a final score. Learning to live together, taming motor aggressiveness, educating sustainability, and learning to compete and not to compare oneself with others are values of maximum interest for the society of the 21st century.

We have tried to show that the legacy offered by informal playful education, which corresponds to the genuine nature of traditional games, should be taken advantage of by formal education. Emotional physical education should make use of the fundamental learning contained in the TSG as intangible cultural heritage (Parlebas, 2016).

Games are above all a source of pleasure, fun, and well-being. For this reason, the distinctive features of the TSG have led their people to acquire learning that is as deep as enjoyable (Etxebeste, 2012; Lavega-Burgués and Navarro-Adelantado, 2015). Parlebas (1969) already advanced that affectivity is the key to the motor conducts of the participants in any game. Thirty years later, this study reaffirms that one of the main contributions of the TSG is their affective dimension, as the capacity to construct emotional communities in any society.

It is under these conditions that TSG directly intervene in the social construction of emotional communities (Rosenwein, 2006). Empathy and respect for the other actors in the game educate the affective sense of feeling members of the same community (McMillan and Chavis, 1986).

## Data Availability Statement

The raw data supporting the conclusions of this article will be made available by the authors, without undue reservation.

## Author Contributions

AC, PL-B, JM-L, and MP: substantial contribution to study conception and design. AC, PL-B, and JM-L: preparation of the document for approval by the Ethics Committee. AC, PL-B, VM-A, SD-S, RL-P, and CS-S: preparation and participation in the empirical work and discussion of data analysis strategies. AC, PL-B, MP, VM-A, SD-S, RL-P, and CS-S: preparation of the database (all variables). AC, PL-B, JM-L, MP, VM-A, and SD-S: database revision. All authors contributed to the article and approved the submitted version.

## Conflict of Interest

The authors declare that the research was conducted in the absence of any commercial or financial relationships that could be construed as a potential conflict of interest.
